# Prevalence of neurological diseases associated with cervical pain and/or signs of cervical myelopathy in French bulldogs: a retrospective analysis of 105 cases

**DOI:** 10.3389/fvets.2024.1431358

**Published:** 2024-07-03

**Authors:** Michele Capasso, Sara Canal, Federica Balducci

**Affiliations:** Anicura I Portoni Rossi Veterinary Hospital, Zola Predosa, Bologna, Italy

**Keywords:** French bulldog, cervical myelopathy, MRI, cervical pain, IVDH, syringomyelia, ganglioneuritis

## Abstract

**Introduction:**

French bulldogs can be affected by several neurological diseases, with myelopathies representing the most frequent cause of neurological signs. Studies focusing on the epidemiology of cervical diseases in this breed are lacking. This study aims to describe the prevalence of neurological pathologies responsible for cervical pain and/or signs of cervical myelopathy, assuming that intervertebral disc herniation represents the most common disease; a second aim was to evaluate how often different pathologies coexist in this spinal region in French bulldogs.

**Materials and methods:**

A retrospective analysis of medical records from the database of a single referral center (AniCura I Portoni Rossi Veterinary Hospital—Zola Predosa, Bologna, Italy) was performed, including French bulldogs presented for neck pain and/or neurological deficits consistent with cervical myelopathy. Clinical and imaging data were reviewed and used as inclusion criteria. Based on the number of MRI-diagnosed diseases, the eligible dogs were divided into three groups.

**Results:**

One hundred five French bulldogs met the inclusion criteria. The most commonly diagnosed condition was an intervertebral disc herniation (66.9%), followed by C2 idiopathic hypertrophic ganglioneuritis (15.1%), cervical syringomyelia (11.5%), congenital osseous malformations (1.4%), spinal arachnoid diverticula (1.4%), neoplasms (1.4%), steroid-responsive meningitis-arteritis (0.7%), traumatic vertebral fractures (0.7%), and other (0.7%). For the vast majority of dogs (75/105) a single pathology was diagnosed, with intervertebral disc herniations accounting for 86.7% of cases, involving C3–C4 IVD most commonly. In the remaining 30 dogs, two or three different and concurrent diseases were detected. Among these 30 dogs, intervertebral disc herniations still remained the most diagnosed condition, in combination with C2 idiopathic hypertrophic ganglioneuritis and syringomyelia in 19 and seven cases, respectively.

**Conclusion:**

The results of this study highlight that different pathologies can affect the cervical spinal cord in French bulldogs, with intervertebral disc herniations representing the most frequent condition, as previously described in the veterinary literature. In almost a third of cases, different pathologies can coexist at the cervical level. However, for cases in which different pathologies are present at the same time, it is not always possible to clearly establish their clinical significance.

## 1 Introduction

The French bulldog (FB) is a canine breed that has experienced a significant increase in popularity in the past decade. In Italy, there have been 4,516 new Italian Kennel Club (ENCI) registrations in 2021, compared with 2,266 in 2016 (Ente Nazionale Cinofilia Italiana; homepage: https://www.enci.it/libro-genealogico/razze/bouledogue-francese). Accordingly, due to the high prevalence of several diseases reported in this breed, strictly related to the dogs’ brachycephalic and chondrodystrophic conformation (1, 2), the number of FBs presented at our institution for any health issue has increased. A recent study focusing on the prevalence of neurological disorders has found that myelopathies are the most frequent cause of neurological signs in this breed, affecting 64.7% of dogs in the study population, followed by encephalopathies, unclassified conditions, and peripheral nervous system (PNS) and muscle disorders (3). Among the causes of myelopathy, intervertebral disc herniation (IVDH), more specifically intervertebral disc extrusion (IVDE), was the most common condition, affecting the cervical vertebral column in nearly 40% of dogs (3). Cervical pain was present in almost 83% of FBs affected by a cervical IVDE; data about the presence of pain related to other cervical spinal cord diseases in this breed are not available (3). In the same study, 2.6% of FBs were diagnosed with multiple neurological conditions; however, these figures concern animals presented for clinical signs at any localization in the central nervous system (CNS) or PNS (3).

FBs can be affected by several pathologies involving the cervical spinal cord (3–6). However, how often different pathologies coexist at the cervical level in this breed has never been investigated. The aim of our study was to retrospectively describe the prevalence of neurological disorders in a large cohort of FBs presented at our institution for neck pain and/or clinical signs suggestive of cervical myelopathy. We hypothesized that IVDE was the most common disease associated with this clinical presentation, with a predilection for the C3–C4 intervertebral disc space (IVDS), as previously reported (3, 4). The second aim of the study was to report the prevalence of multiple diseases simultaneously affecting the cervical spinal cord in this breed.

## 2 Materials and methods

The medical records of FBs presented at AniCura—I Portoni Rossi Veterinary Hospital between April 2018 and April 2023 for neck pain and/or cervical myelopathy were retrospectively reviewed. Dogs were included if they underwent a complete neurological examination, consistent with a C1-T2 spinal cord segment neuroanatomical localization, and magnetic resonance imaging (MRI) of the cervical vertebral column. Further diagnostic examinations could include one or a combination of the following: spinal radiographs, cerebrospinal fluid (CSF) analysis, or histopathology. Dogs were excluded if the medical records or imaging studies were incomplete or not available. Dogs with clinical signs suggestive of concurrent intracranial or T3-S3 spinal cord segment involvement were also excluded.

Collected data included signalment (age, sex, body weight), onset of clinical signs, neurological examination, and MRI findings. When the CSF was analyzed, the collection site and results were also reported. If histopathological examinations were performed, the results were reported.

The onset was defined as the time between the beginning of clinical signs and the presentation at our institution; it was classified as acute (<48 h), subacute (3–7 days), or chronic (>7 days) (7).

All dogs underwent a neurological examination performed by either a board-certified neurologist (FB, SC) or a neurology resident (MC).

The neurological status was graded on a scale from 1 to 4 scores, modified from Schmied et al. (8). Grade 1 included dogs with cervical hyperesthesia (reported by the owner or detected during neurological examination) without neurological deficits or lameness. Grade 2 included dogs with forelimb lameness (monoparesis) or ambulatory tetra/hemiparesis. Grade 3 was defined as non-ambulatory tetraparesis or tetraplegia with intact nociception. Tetraplegic dogs with respiratory impairment/absent deep pain perception were assigned to the Grade 4 group. In Groups 2, 3, and 4, cervical pain was also recorded, when present.

All MRI studies were performed with a 1.5 Tesla scanner (Vantage Elan, Canon Medical Systems Europe B.V.) under general anesthesia and included a minimum of T2- and T1-weighted images on both sagittal and transverse planes. Additional sequences were acquired at the radiologist’s discretion to ensure a confident diagnosis.

All MRI studies were reviewed by both board-certified neurologists (FB, SC) using a DICOM viewer (2020 Horos Project TM), and the decision on the diagnosis was reached on a consensus basis. Dogs were assigned to one or more of the following disease categories: C2 idiopathic hypertrophic ganglioneuritis (C2-IHGN); steroid-responsive meningitis-arteritis (SRMA); spinal trauma (ST); syringomyelia (SM); congenital osseous malformations (COMs) other than Chiari-like malformation (CLM); spinal arachnoid diverticulum (SAD); cervical spinal cord neoplasms (CNs); IVDH, including IVDEs, intervertebral disc protrusions (IVDPs), and hydrated nucleus pulposus extrusions (HNPEs); and other (e.g., cyst-like lesion in the fourth ventricle).

A C2-IHGN was diagnosed when, at the level of the C2 spinal cord segment, a severe focal enlargement of one or both spinal nerve roots was identified; the enlarged spinal nerve roots showed T2W hyperintensity and T1W isointensity, with strong contrast enhancement. Additional imaging features were a triangle-shaped spinal cord on the transverse sections due to the compression exerted by the roots in the bilateral form, and a focal area of intramedullary hyperintensity at the level of compression or spinal cord central canal dilation (9).

Cervical SM was diagnosed when a longitudinal elongated T2W hyperintense, T1W hypointense, and FLAIR-suppressed cavitation of the cervical spinal cord was detected (10). In the cases diagnosed with SM and for which the MRI study included the caudal fossa and the craniocervical junction (CCJ), the abnormality believed to be responsible for the SM was described, similarly to a previous study (5). The maximum diameter and symmetry of the syrinx were evaluated on T2W transverse images, when available (5). When a focal dilation of the central canal was detected in correspondence with a site of spinal cord compression exerted by a bilateral C2-IHGN, it was not classified as an SM (9).

An SAD was defined as a focal tear-drop-shaped dilation of the subarachnoid space, well identifiable with the HASTE sequences, with a T2W hyperintense, T1W hypointense, and/or FLAIR hypointense signal (11).

The differentiation between IVDE and IVDP was based on MRI features, as proposed for thoracolumbar IVDEs (12). An HNPE was diagnosed when homogeneously hyperintense T2W epidural material [isointense in all sequences up to the underlying intervertebral disc (IVD)] was identified ventral to the spinal cord, in correspondence with a narrowed IVD, associated or not with T2W intramedullary hyperintensity (13).

MRI criteria used for a diagnosis of SRMA, COM, spinal trauma, and presumed cervical spinal neoplasm were based on the features previously reported in the veterinary literature (14–22).

According to the MRI findings and presumptive diagnoses, dogs were divided into three groups based on the number of disease categories. Group 1 included dogs with only one detected pathology; dogs with multiple IVDEs or IVDPs or both IVD extrusion and protrusion were included in this group. When multiple discs were affected, the herniation site responsible for the clinical signs was identified based on clinical signs and the degree of spinal cord compression (23). Group 2 and Group 3 included dogs with two or three coexisting disease categories, respectively.

An abnormal CSF analysis was determined if the findings exceeded the reference values of ≤5 cells/μL for nucleated cell count and ≤25 mg/dL or ≤45 mg/dL for total protein concentration in the cisternal and the lumbar puncture, respectively. An abnormal cell morphology or distribution was also considered an anomalous finding. Increased total protein levels with normal nucleated cell count were characterized as albuminocytological dissociation (24).

Data were analyzed using a commercially available spreadsheet (Microsoft Excel 2020, Microsoft, Redmond, WA, United States), allowing descriptive statistics to be performed. Variables with a continuous distribution are reported as mean values and ranges, while categorical variables are reported as percentages.

## 3 Results

Between April 2018 and April 2023, a total of 1,321 FBs were presented at AniCura I Portoni Rossi Veterinary Hospital; 503 (38%) of them were referred for a neurological evaluation. Among the neurological cases, 116 dogs (23.1%) were presented for neck pain and/or clinical signs suggestive of cervical myelopathy. Eleven dogs were excluded because they did not undergo an MRI investigation of the cervical spine. One hundred five dogs met the inclusion criteria and were included, representing 7.9% of the whole FB hospital population and 20.9% of FBs presented for neurological evaluation.

The mean age at presentation was 56.5 months (4.7 years), ranging from 6 months to 172 months (0.5–14.3 years). There were 67 male dogs (60 entire and seven neutered males) and 38 female dogs (11 entire females and 27 neutered females). Data about body weight were available in 103/105 dogs. The mean body weight was 12.55 kgs (range 7.5–22.3 kgs).

The onset of clinical signs was classified as acute in 23 dogs (22.1%), subacute in 31 dogs (29.8%), and chronic in 50 dogs (48.1%). Data about the onset of clinical signs were not available for one dog.

Overall cervical pain was reported in 103/105 (98.1%) dogs. Based on their neurological status at the time of presentation, 66 dogs (62.9%) were classified as Grade 1. Thirty-three (31.4%) and six dogs (5.7%) showed a clinical presentation consistent with Grade 2 and Grade 3, respectively. No dogs were included in the group of tetraplegia with respiratory impairment or deep pain perception loss (Grade 4).

Considering the overall population of 105 dogs, a pathological condition of the cervical spine was detected 139 times. Based on MRI findings, we have diagnosed nine different pathologic entities: IVDH 93/139 (66.9%), C2-IHGN 21/139 (15.1%), SM 16/139 (11.5%), COMs 2/139 (1.4%), SAD 2/139 (1.4%), intra- or extra-medullary neoplasia 2/139 (1.4%), SRMA 1/139 (0.7%), cystic lesion in the fourth brain ventricle 1/139 (0.7%), and traumatic myelopathy 1/139 (0.7%).

Based on the number of diseases identified on MRI scans, dogs were assigned to three groups: Group 1 included 75 dogs, and Group 2 and Group 3 included 26 and four dogs, respectively.

In Group 1, the most common disease was IVDH, affecting 65/75 dogs (86.7%), for a total of 85 involved IVDs. The most commonly affected IVD was C3–C4 (37/85), followed by C4–C5 (19/85), C2–C3 (19/85), C5–C6 (7/85) and C6–C7 (3/85).

In 47/65 dogs of this group, the clinical signs were attributable to a single IVDE. In three cases, two IVDEs were detected. Fifteen dogs had a diagnosis of IVDE and coexisting IVDP; in no case was an IVDP diagnosed as the only pathology.

**Figure 1:**
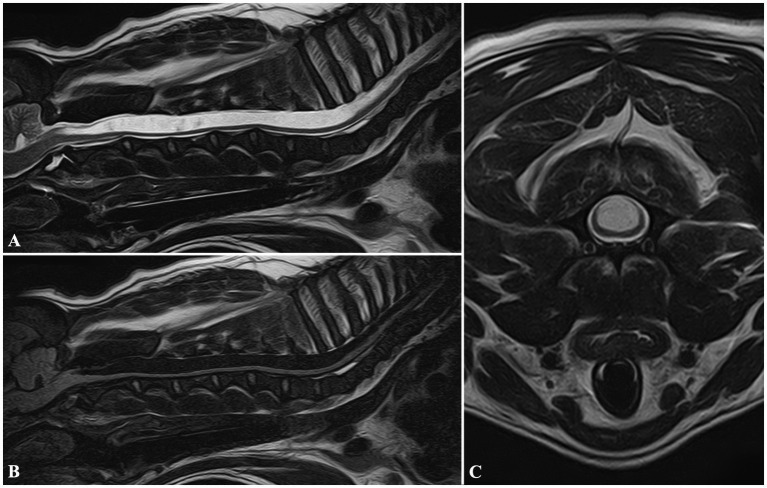
MRI images of a FB included in Group 1, diagnosed with cervical syringomyelia. Sagittal T2W **(A)**, and FLAIR **(B)** images showing a wide cavitation involving the spinal cord parenchyma, and extending from C2 to T8 vertebrae. Transverse T2W **(C)** image at the level of the C3 vertebral body showing the maximum diameter of the lesion, symmetrically located, and involving more severely the dorsal half of the spinal cord.

In Group 1, 5/75 dogs were affected by SM: the mean syrinx diameter was 6.66 mm (range 3.76–10 mm). In all the cases, SM affected the dorsal horns of the spinal cord symmetrically (Figure 1). Furthermore, a CLM was found in all dogs, with concurrent C1–C2 dorsal compression in 2/5 dogs. The following other diseases were found: two SADs, one SRMA, one COM, and a cyst-like lesion in the fourth ventricle (Table 1).

**Table 1:** Signalment, history, clinical signs, and distribution of cervical diseases in 75 FBs of Group 1.

Disease	Number of cases (%)	Disease location	Mean age* (range)	Onset	Cervical pain no of cases (%)	Neurological grade
• IVDH	65/75 (86.7%)		54.5 (19–102)	A: 15/65 S: 20/65 C: 30/65	65/65 (100%)	1: 43/65 2: 18/65 3: 4/65
		IVDE: 47/65 C2-C3: 9 C3-C4: 25 C4-C5: 11 C5-C6: 2				
		Double site IVDEs: 3/65 C2–C3 + C3–C4 C2–C3 + C5–C6 C4–C5 + C6–C7				
		IVDE + (IVDP): 15/65 C2–C3: 1 + (P) 7 C3–C4: 8 + (P) 3 C4–C5: 3 + (P) 4 C5–C6: 3 + (P) 1 C6–C7: 0 + (P) 2				
• SM	5/75 (6.7%)	Extended between VBs: from C3 to C4: 1/5 from C3 to T2: 2/5 from C3 to T5: 1/5 from C3 to T8: 1/5	29 (6–49)	A: 1/5 S: 1/5 C: 2/5 N/A: 1/5	5/5 (100%)	1: 4/5 2: 1/5
• SAD	2/75 (2.7%)	C1–C2 (1) C2–C3 (1)	58 (9–107)	A: 1/2 S: 1/2	2/2 (100%)	1: 1/2 2: 1/2
• SRMA	1/75 (1.3%)	N/A	49	S	1/1 (100%)	1
• COM	1/75 (1.3%)	Malformed left occipital condyle	7	C	1/1 (100%)	1
• Other	1/75 (1.3%)	Cyst-like lesion within the fourth ventricle	10	A	1/1 (100%)	1

A, acute; C, chronic; COM, congenital osseous malformation; IVDE, intervertebral disc extrusion; IVDH, intervertebral disc herniation; N/A, not applicable; (P), protruded disc; S, subacute; SAD, spinal arachnoid diverticulum; VBs, vertebral bodies; SM, syringomyelia; SRMA, steroid responsive meningitis-arteritis. *Ages are expressed in months.

**Figure 2:**
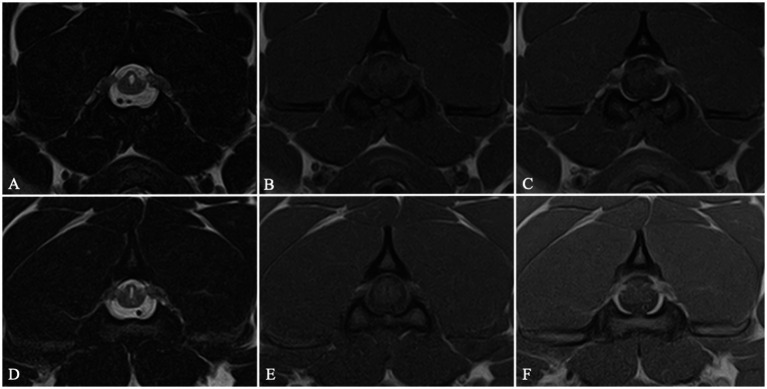
MRI transverse T2W **(A,D)**, T1W **(B,E)** and T1W post-contrast medium administration **(C,F)** images at the level of C2 vertebral body showing a bilateral C2 idiopathic hypertrophic ganglioneuritis in 2 dogs belonging to Group 2. C2 nerve roots appear bilaterally enlarged and contrast-enhancing. The spinal cord is distorted and exhibits a triangular shape on cross-sectional images, with focal secondary enlargement of the dorsolateral subarachnoid space.

Group 2 included 26 dogs with two distinct pathological conditions. IVDE was the most commonly diagnosed concurrent disease, affecting 24/26 dogs for a total of 27 IVDs: C3–C4 (9/27), C4–C5 (8/27), C2–C3 (5/27), C5–C6 (2/27) and C6–C7 (3/27). C2-IHGNs were diagnosed in 20 FBs: in 18/20 dogs, the disease affected both C2 nerve roots (Figure 2), while two cases showed a monolateral nerve root involvement (one right, one left).

**Figure 3:**
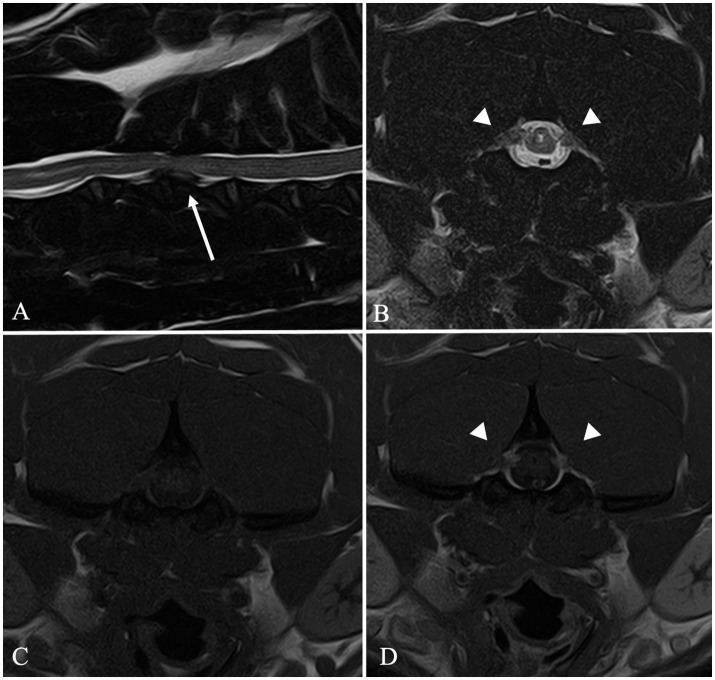
MRI images of a FB included in Group 2. Sagittal T2W image **(A)** shows a C3–C4 intervertebral disc extrusion (arrow). Transverse T2W **(B)**, T1W **(C)** and T1W post-contrast medium administration **(D)** images at the level of C2 showing a bilateral C2 idiopathic hypertrophic ganglioneuritis. C2 nerve roots appear bilaterally enlarged (arrowheads) and contrast-enhancing **(D)**.

In 18 cases, a diagnosis of concurrent IVDH and C2-IHGN was made (Figure 3), including a case of HNPE and three cases of double-site IVDE. Six out of 26 dogs showed a concurrent IVDE and SM. Finally, in two dogs, a cervical SM and an C2-IHGN were identified (Table 2).

**Table 2:** Signalment, history, clinical signs, and distribution of cervical diseases in 26 FBs of Group 2.

Associated disease	Number of cases (%)	IVDH sites	Mean age* (range)	Onset	Cervical pain no of cases (%)	Neurological grade
• IVDH + C2-IHGN	18/26 (69.3%)		63.7 (24–107)	A: 2/18 S: 6/18 C: 10/18	18/18 (100%)	1: 10/18 2: 7/18 3: 1/18
		IVDE + C2-IHGN: 15/18 C2–C3: 3 C3–C4: 5 C4–C5: 5 C5–C6: 2				
		Double site IVDEs + C2-IHGN: 3/18 C2–C3 + C3–C4 C2–C3 + C6–C7 C3–C4 + C4–C5				
• IVDH + SM	6/26 (23%)	C3–C4: 2 C4–C5: 2 C6–C7: 2	63.6 (47–83)	A: 1/6 S: 2/6 C: 3/6	5/6 (83.3%)	1: 3/6 2: 3/6
• C2-IHGN + SM	2/26 (7.7%)	N/A	75 (both 75 m)	A: 1/2 C: 1/2	2/2 (100%)	2 1

A, acute; C, chronic; C2-IHGN, C2 idiopathic hypertrophic ganglioneuritis; IVDE, intervertebral disc extrusion; IVDH, intervertebral disc herniation; N/A, not applicable; S, subacute; SM, syringomyelia. *Ages are expressed in months.

In eight cases, SM was identified. In 7/8 dogs, the MRI study included the cranial caudal fossa and identified a CLM in 7/7 dogs. Transverse images for the evaluation of the maximum diameter and symmetry of the syrinx were available in 5/8 dogs. The mean syrinx diameter was 4.64 mm (range 2.2–7.1 mm). The syrinx was symmetrically located in the center or in the dorsal half of the spinal cord in 2/5 and 3/5 dogs, respectively.

Group 3 included four dogs; in all the cases, an IVDH was observed, for a total of seven IVDs. Other diseases were SM (3/4), neoplasms (2/4), COM (1/4), bilateral C2-IHGN (1/4), and vertebral traumatic lesions (1/4). In 2/3 SM cases, the mean syrinx diameter was estimated (4.94 mm, range 3.4–6.48 mm); the syringes symmetrically involved the dorsal horns. In one case, a CLM was also present (1/2). The frequency with which these findings were associated with each other is shown in Table 3.

**Table 3:** Signalment, history, clinical signs, and distribution of cervical diseases in 4 FBs of Group 3.

Associated disease	Age (months)	Onset	Cervical pain (Y/N)	Neurological grade
• C4–C5 IVDE + SM + C3–C4 block vertebra	40	C	Yes	2
• C3–C4 IVDE + SM + C2-IHGN	69	C	Yes	1
• C3–C6 IVDP + C2 extramedullary neoplasm + traumatic fractures of C4–C7 vertebral processes	172	A	Yes	3
• C4–C5 IVDP + SM + C2 intramedullary neoplasm	62	C	No	2

A, acute; C, chronic; C2-IHGN, C2 idiopathic hypertrophic ganglioneuritis; IVDE, intervertebral disc extrusion; IVDP, intervertebral disc protrusion; SM, syringomyelia.

Cerebrospinal fluid, collected at the level of the cerebellomedullary cistern, was analyzed in three dogs. In two cases (one with an IVDH with mild spinal cord compression and one with a cyst in the fourth ventricle), CSF analysis was performed to rule out inflammatory-infectious pathology. In both cases, the CSF was unremarkable. The third case, belonging to Group 3 (concurrent C4–C5 IVDP, C2–C5 SM, and C2 intramedullary suspected neoplasm), had a mild mononuclear cell pleocytosis (9 nucleated cells/mcL) and normal protein content.

**Figure 4:**
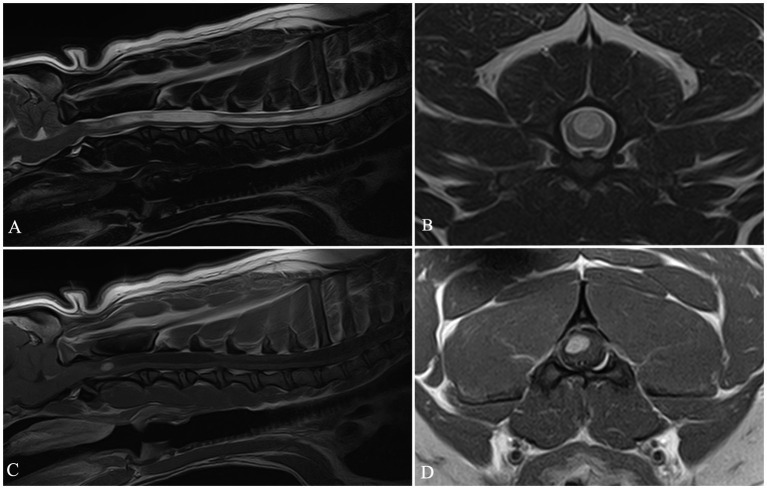
Sagittal T2 W image **(A)**, transverse T2 W image **(B)**, sagittal and transverse T1W post-contrast images **(C,D)**. In this dog a mild C4–C5 intervertebral disc protrusion **(A)**, cervical syringomyelia **(A–C)** and C2 intra-axial neoplasm **(C,D)** were diagnosed.

Histopathology was performed in two cases. In the first dog, belonging to Group 1, a final histopathological diagnosis of SRMA was made. In the second case (Figure 4), belonging to Group 3, a surgical biopsy was submitted for histopathological examination, with a final diagnosis consistent with spinal cord intra-axial hemangioblastoma.

## 4 Discussion

In our study, FBs presented for neurological complaints represented 38% of the entire FBs population referred to our institution over a 5 year period (April 2018–April 2023). This figure is significantly higher than that in a previous study (3), which reported that 18.7% of FBs were presented for neurological clinical signs in a 14 year period (January 2002–January 2016). Both studies were performed at veterinary referral centers, although in different time periods. We can speculate that such different percentages might be attributable to both the increased diffusion of this breed and the strong genetic selection in favor of brachycephalic and chondrodystrophic characters in most recent years (1, 2). However, we cannot rule out that these differences may indicate variations in prevalence across geographical and genetic populations; additionally, they might be linked to the different inclusion criteria.

In our study period, 105 FBs underwent advanced imaging due to signs consistent with neck pain and/or cervical myelopathy, representing 7.9% of the general FB hospital population presented for any health issue and 20.9% of FBs admitted at our institution for neurological complaints. Although myelopathies represent the most common cause of neurological disorders in this breed (3), studies focusing on the prevalence of cervical neurological diseases in FBs are lacking.

Cervical hyperesthesia was a critically important feature in our cohort of dogs, as it was present in nearly all dogs (103/105). Furthermore, neck pain represented the only clinical complaint in 61.7% of FBs enrolled in the study population.

Causes that can lead to cervical hyperesthesia are categorized as mechanical (due to lesions involving the cervical vertebral column or its supporting structures), neuropathic (due to lesions of the somatosensory system involving the spinal cord, the dorsal root ganglia, or the spinal nerves), or other conditions not directly related to the cervical region (25).

In 102 out of 103 dogs, we found that the neck pain was caused by a pathological condition affecting the cervical spinal cord and/or the vertebral column and its supporting structures, while in 1/103 dogs the MRI failed to reveal abnormalities in the cervical region. This single case involved a 10 month-old male FB, presented for an acute onset of reluctance to move and be touched and with an antalgic posture of the head. The results of neurological examination were within normal limits, except for a low head carriage manifested after neck manipulations. The MRI showed a cyst-like lesion involving the fourth ventricle. The CSF analysis was unremarkable. Cervical hyperesthesia associated with intracranial disease has been reported in the veterinary literature as a consequence of intracranial pathologies responsible for increased intracranial pressure and stretching of the meninges (26). In a recent study focusing on the epidemiology of cervical hyperesthesia, neck pain was related to brain diseases in 2.2% of cases (three dogs with intracranial neoplasia and one dog with hydrocephalus) (27). In our case, the cervical pain might be linked to localized alterations in the CSF flow within the fourth ventricle and foramen magnum resulting from the cyst. This hypothesis is supported by a case report detailing comparable clinical signs and MRI findings in a dog diagnosed with a choroid plexus cyst within the fourth ventricle (28).

The most common disease in our population was IVDH, diagnosed in 88.6% (93/105) of dogs included in the study. Moreover, an IDVH was a concurrent spinal disease in 92.3 and 100% of dogs belonging to Group 2 and Group 3, respectively. This finding aligns with the previous literature, indicating that IVDHs are the primary cause of myelopathy in FBs (3), related to the chondrodystrophic conformation of this breed (2). Concerning the type of IVDH, 82.3% were extruded IVDs (including 97 Hansen Type I IVDEs and one HNPE), and 17.7% IVDPs. Furthermore, IVDPs were always diagnosed together with IVDEs or other diseases, preventing the evaluation of their clinical relevance.

Among the 91 dogs with cervical IVDE, the mean age at presentation was 57.4 months (4.7 years), ranging from 19 to 107 months (1.5–8.9 years), in line with the literature on this breed (3, 4). FBs appear to have a lower mean age of onset of IVDE than the median age of 7.8 years in Dachshunds and other chondrodystrophic breeds (29–32). In the study of Mayousse et al., it was hypothesized that this difference could depend on earlier chondroid degeneration of IVDs in the FBs than in other chondrodystrophic dogs, but further investigations are necessary to establish a real difference in the IVDs degenerative processes between predisposed breeds (3).

An IVDE was diagnosed as the sole disease and was considered responsible of cervical hyperesthesia in 63.1% of dogs presenting with neck pain. This finding aligns with a previous study investigating the causes of cervical pain in dogs, in which IVDEs accounted for 64.9% of cases (27). The role of IVDEs in causing cervical pain in FBs in our study might be underestimated, as dogs with IVDEs belonging to Groups 2 and 3 were excluded. In fact, for these dogs, IVDEs remain the most prevalent pathology; however, while they may potentially be the primary cause of pain, we cannot establish the role that concurrent pathologies play in generating cervical hyperesthesia.

The neurological grade at presentation in dogs affected by IVDEs was Grade 1 in 62.7% of dogs (57/91), and Grade 2 in 31.9% (29/91) of dogs; just 5.5% (5/91) of dogs were not ambulatory (Grade 3). These data are consistent with what is reported in the literature, indicating that the clinical presentation of dogs affected by cervical IVDEs is generally less severe than that of dogs affected at the thoracolumbar level, regarding both anatomical and pathological issues. The vertebral canal in the cervical region is wider, providing more epidural space surrounding the cervical spinal cord, than the vertebral canal in the thoracolumbar region; furthermore, a less massive inflammatory response has been demonstrated in the cervical epidural space following an IVDE (33–35). Another possible anatomical explanation is a significant difference in the longitudinal extent of extruded IVD material between cervical and thoracolumbar IVDEs in this breed, with this material being significantly less spread in cases affected by cervical IVDEs (4). Such differences have been hypothetically explained by a more robust meningovertebral ligament in the cervical area than in the thoracolumbar region (36) or by a potential high-energy and high-velocity modality of extrusion of the thoracolumbar IVDs (4).

Regarding the overall number of IVDEs, the most frequently affected IVD was C3–C4 (46.3%), followed by C4–C5 (26.3%), C2–C3 (16.8%), C5–C6 (7.4%) and C6–C7 (3.2%), in agreement with two recent studies specifically focused on the FBs (3, 4).

One dog was diagnosed with an HNPE; it was a 7 year-old male who presented for cervical hyperesthesia. Age of onset was higher than in dogs affected by Hansen Type I IVDEs, consistent with the literature (13, 37, 38). Although the prevalence of this type of disc herniation across different dog breeds is not well defined, some breeds appear to be over-represented in the literature. These include Yorkshire Terriers, Miniature Pinschers, and Beagles (13, 37–41). Notably, no cases of cervical HNPE have been reported in FBs.

Although this pathology is described in both non-chondrodystrophic and chondrodystrophic breeds (42), the low prevalence in the study population could be explained by the chondroid disc degeneration affecting this breed.

The results of our study show that a presumed C2-IHGN is a frequent finding in FBs undergoing MRI examination of the cervical spinal cord. It was detected in 20% of our study population and is therefore the second most prevalent disease in our dogs. Hypertrophic inflammatory changes in the cervical spinal nerves are well recognized in humans and are commonly related to a chronic inflammatory demyelinating polyradiculoneuropathy (CIDP) (43). In dogs, this condition can be diagnosed both as an incidental finding and as a cause of clinical signs of cervical myelopathy (9). In the study of Joslyn et al. the largest cohort of dogs, including twelve dogs affected by the bilateral form, was described (9). Of note, nine out of 12 cases were American Staffordshire terrier dogs, and no FBs were present in the study. In this study, in four out of 12 cases (33%), the disease was considered not clinically significant, as concomitant with idiopathic epilepsy and cervical IVDEs (9).

In our study, suspected C2-IHGN was never diagnosed as the sole pathological entity, precluding the opportunity to evaluate its clinical relevance. Eight dogs had a concurrent surgically treated IVDE, and all of them regained a normal neurological status in 14 to 30 days after surgery; therefore, for these dogs, the C2-IHGN was considered an incidental finding, as in several previously reported cases (9). For the other medically managed cases (both IVDHs or SM) the clinical significance of the coexisting C2-IHGN cannot be clearly established.

Data about age, onset, and neurological grade were not discussed for dogs diagnosed with this condition; in fact, C2-IHGN represented a concurrent diagnosis in all cases and was considered an incidental finding in several cases, probably having little or no impact on these clinical data. Further studies are needed to confirm our hypothesis.

Cervical SM is commonly diagnosed in canine brachycephalic toy breeds; furthermore, FBs have skull conformation features similar to those of other breeds with an overrepresentation of this disease, such as the Cavalier King Charles spaniel (CKCS) and Griffons de Bruxelles (GB) (44). In our study population, cervical SM was detected in 16 dogs (15.2%); moreover in 5/105 (4.8%) cases it was diagnosed as the sole disease affecting the cervical spinal cord. In our study, the prevalence of SM in FBs was higher than the 8% previously reported (5); this may suggest possible variations in prevalence across different regions of the world and genetic pools.

The mean age of dogs diagnosed with SM alone was 28.8 months (2.4 years), ranging from 6 to 49 months (0.5–4.1 years), which is lower than the mean age of dogs affected by IVDH, although a reliable comparison between the two groups cannot be made due to the small number of animals affected by SM. In these five dogs, cervical hyperesthesia was consistently present, and 1/5 additionally displayed lameness and proprioceptive deficits in one front limb. In all cases, pain was manifested as yelps, cervical rigidity and low head carriage, and could be related to the width of the syrinx and the involvement of the dorsal horns, as previously stated (45–47). Nevertheless, no dogs exhibited phantom scratching, in accordance with a previous study focused on the clinical significance of SM in FBs (5). However, phantom scratching is not consistently present in all dog breeds affected by symptomatic SM, as stated for GBs (48). In the CKCS, a study investigated the association between phantom scratching and MRI characteristics of the syrinx. It was found that involvement of the C3–C6 spinal segment dorsal horns was significantly associated with this clinical sign (49). Four out of 5 FBs in our study and three out of four dogs in the study of Ricco et al. had these MRI characteristics of the syrinx, despite the absence of phantom scratching (5). Therefore, additional research with a larger cohort of FBs is needed to determine whether phantom scratching could be a clinical sign in FBs affected by cervical SM and whether the pathogenetic mechanism of phantom scratching hypothesized for the CKCS could apply to FBs.

In the remaining 11 dogs, cervical SM was found alongside one or more additional diseases. Consequently, the clinical significance of SM in this subgroup of dogs remains unclear. This ambiguity arises from the presence of concurrent pathological conditions in the cervical region and the well-documented occurrence of asymptomatic forms of SM (45).

For 15 out of 16 dogs diagnosed with cervical SM, the cranial caudal fossa was also included in the MRI investigation. In 14 dogs, a CLM was detected, which in two cases was associated with a concurrent C1–C2 dorsal spinal cord compression. These results reflect what is reported in the literature: although Chiari-like malformation is the most commonly detected disease associated with cervical SM in dogs (especially in the CKCS breed), other concurrent cranio-cervical junction anomalies (atlanto-occipital overlapping, occipitoatlantoaxial malformations, atlantoaxial dural bands) are commonly associated with the development of this spinal cord condition (5, 50).

One dog was diagnosed with a C2 spinal cord intra-axial hemangioblastoma and concurrent SM extending from the C2 to the C5 spinal cord segments. The MRI study included the cranial caudal fossa, and a CLM was also detected. Therefore, it is impossible to establish whether the two findings may have an etiopathogenetic correlation or whether they represent two different coexisting pathologies. However, we hypothesized that the presence of an intra-axial tumor may have played a role in the formation of the syrinx, similarly to what is described in human medicine about the association between spinal hemangioblastomas and SM (51).

Although bony malformations, both symptomatic and asymptomatic, are extremely common in the thoracolumbar region of FBs (17, 18), they are rarely reported at the cervical level (52). Congenital osseous malformations other than CLM were detected in 2/105 (1.9%) dogs. One dog had a C3–C4 vertebral body fusion (block vertebra), with concurrent SM and C4–C5 IVDH. In this case, the COM was unlikely to be directly associated with the clinical signs. However, it played an important role in generating biomechanical alterations in the cervical spine, thereby predisposing the dog to degenerative changes in the adjacent IVD, as previously described (53). In the other dog, the bony malformation affecting the left occipital condyle was the direct cause of the patient’s clinical presentation, due to foramen magnum stenosis.

An SAD was diagnosed in two out of 105 dogs. The FB is overrepresented for SAD, which is the second most common cause of myelopathy in this breed (3). However, SAD in FBs is preferentially located in the thoracolumbar region and is frequently correlated with vertebral malformations or concurrent spinal cord disorders (54). Therefore, our data are in agreement with a previous study detecting SADs at the cervical level in just three cases out of 222 suffering from myelopathy (3).

A neoplastic disease was diagnosed in two dogs, representing 1.9% of the entire study population, thus suggesting that this condition was an uncommon cause of clinical signs of cervical myelopathy in our group of dogs. A similar low prevalence has already been reported before, regarding both spinal and vertebral neoplasia (3).

One dog was diagnosed with an SRMA. The data regarding such a low number of immune-mediated inflammatory diseases should be interpreted with caution. Indeed, our results could have been heavily influenced by the inclusion criteria, which excluded animals with multifocal clinical signs. This could have significantly reduced the number of included cases with CNS inflammatory pathologies, as exclusive involvement of the cervical spinal cord is less common than the concurrent involvement of the brain (55).

Data emerging from our study highlight that in the majority of FBs the clinical signs are related to a single disease. However, in almost a third (30.8%) of dogs, two or three concomitant pathologies coexist. This potentially complicates determining which pathology or pathologies are causing the clinical signs, or whether they may represent incidental findings. A previous study reported the presence of multiple neurological diseases in nine out of 343 FBs included (3). However, in these cases, neither the location nor the type of the diagnosed pathologies was discussed, preventing a direct comparison with our results.

In FBs, multiple simultaneous neurological diseases affecting the thoracolumbar spine are documented (54, 56). Nevertheless, similar studies focusing on the cervical region in FBs are lacking. In the study of Olender et al., which investigated cervical jerks as a sign of neck pain or cervical myelopathy, 13 out of 20 dogs were FBs; 5/13 had a diagnosis of multiple-site IVDE but no other concurrent pathological findings were detected (57). In the study of Ricco et al., the significance of SM in 12 FBs was investigated (5). In this study, it was found that 8/12 had a CLM and 4/12 a dorsal compression of the spinal cord at the C1–C2 level. Within these two groups, three and two dogs, respectively, were also diagnosed with a concurrent cervical IVDH. Assessment of syrinx features did not help to predict the presence of pain in the affected FBs; conversely, all painful FBs were also affected by an IVDH. The authors concluded that pain encountered in these FBs was probably related to disc disease (5).

In our study population, concurrent IVDH and SM was one of the most common combinations of diseases. This finding can be explained again by this breed’s brachycephalic and chondrodystrophic conformation, predisposing the dogs to CLM and secondary SM, and chondroid degeneration of IVDs, respectively (1, 2, 48, 58, 59).

In our study, another common combination of diseases involved IVDHs and C2-IHGN. In most cases, this latter was considered an incidental finding. Furthermore, to the best of the authors’ knowledge, C2-IHGN has never been described before in this breed. Therefore, further investigations are necessary to establish a possible genetic predisposition and the true clinical relevance of this disease in FBs.

It should be noted that the high prevalence observed in our study could be due to random factors. Typically, the sagittal T2W sequence is the first to be acquired in a cervical MRI study, but C2-IHGN is better visualized on transverse images at the level of the C1-C2 intervertebral foramina (9).

In our study population, several MRI features visible on T2W sagittal images guided us to investigate this area and identify C2-IHGN. These features included focal subtle central canal dilation at the C2 level (found in 14 out of 20 cases), parasagittal enlargement of the C2 nerve roots (found in 9 out of 20 cases), and the presence of syringomyelia extending to the C2 segment (found in 2 out of 20 cases).

In contrast, in the study by Joslyn et al., features such as central canal dilation or T2W hyperintensity of the spinal cord were not detected in 4 out of 12 dogs (9). Therefore, in the authors’ opinion, the combination of minimal or absent alterations on sagittal MRI images, along with the concurrent presence of clinically significant pathologies with more prominent MRI findings, may contribute to the underestimation of C2-IHGN.

However, whether the prevalence described in our population is related to a higher incidence of the pathology in French Bulldogs or simply influenced by a lower risk of being underdiagnosed in our study remains to be clarified. Further investigations specifically focused on C2-IHGN in this breed are needed to determine the possible genetic predisposition and clinical significance.

Our study has several limitations, primarily due to its retrospective nature. The dogs included were all referred to our hospital by general practitioners. Therefore, the data may be subject to selection bias and may not be representative of the broader population of FBs.

In most cases the diagnosis was based exclusively on imaging findings; CSF analysis and histopathology were available in only 3 and 2 cases, respectively.

Due to the retrospective nature of our study, MRI protocols were not standardized and were adapted on a case-by-case basis. Although a minimum of standard sequences was present in all cases, variations in protocols may have influenced the consistency of the imaging findings. Additionally, in one case of SM, the MRI study did not include an examination of the caudal cranial fossa, and in three cases, the transverse images were not available for evaluation of the maximum diameter and symmetry of the syrinx.

Another significant limitation is the absence of a histopathological examination in cases diagnosed with suspected C2-IHGN. The presumed diagnosis relied solely on imaging characteristics, and the lack of histological confirmation precludes the definitive exclusion of other pathologies involving the nerve roots.

Our study is the first focused on the distribution of neurological diseases responsible for cervical pain or cervical myelopathy in a large cohort of FBs. Our results showed that IVDEs represent the most common pathology, with a preferential localization at the C3–C4 IVD, as previously stated in the literature. Surprisingly, the second most common pathology was suspected C2-IHGN; this finding is new to the literature, in which, to the authors’ knowledge, the FB is not among the studied breeds. However, whether the detection of this alteration in this breed should always be considered clinically significant or an incidental finding remains to be investigated through further research. Cervical SM was diagnosed in 15.2% of dogs, but it was considered responsible for the clinical signs in 4.8% of dogs. Other pathologies, such as SADs, COMs, and neoplasms, were poorly represented.

## Data availability statement

The raw data supporting the conclusions of this article will be made available by the authors, without undue reservation.

## Ethics statement

Ethical approval was not required for the studies involving animals in accordance with the local legislation and institutional requirements because the animal study was a retrospective study. Written informed consent was not obtained from the owners for the participation of their animals in this study because it was a retrospective study on routine clinical work-up.

## Author contributions

MC: Conceptualization, Data curation, Formal analysis, Investigation, Resources, Writing – original draft, Writing – review & editing. SC: Formal analysis, Investigation, Resources, Supervision, Writing – review & editing. FB: Conceptualization, Formal analysis, Investigation, Project administration, Resources, Supervision, Writing – review & editing.
